# Displacive In‐Plane Ferroelectricity with Domain‐Specific Curie Temperature in Van der Waals Semiconductors

**DOI:** 10.1002/advs.202518341

**Published:** 2026-01-04

**Authors:** Peter Sutter, Eli Sutter

**Affiliations:** ^1^ Department of Electrical & Computer Engineering University of Nebraska‐Lincoln Lincoln Nebraska USA; ^2^ Department of Mechanical & Materials Engineering University of Nebraska‐Lincoln Lincoln Nebraska USA

**Keywords:** displacive ferroelectrics, in‐plane superlattice, in situ electron diffraction, tin monochalcogenides, van der Waals semiconductors

## Abstract

Van der Waals bilayers assembled by mechanical stacking of 2D layers have been shown to realize symmetry‐breaking mechanisms, such as sliding‐ or moiré ferroelectricity, which are without equivalent in 3D ferroelectrics. Here, we discuss emergent, highly unconventional behavior in thicker semiconducting few‐layer SnS(e) van der Waals ferroelectrics crystallizing in a distorted polar structure, obtained by bottom‐up synthesis. Electron diffraction at variable temperature demonstrates a gradual displacive transformation between the polar low‐temperature and symmetric high‐temperature phase, with significant variability in both the lattice constants and the Curie temperature (*T*_C_) where the transition is completed. Strikingly, such variations are even found among stripe domains in individual crystals, where lattice constants and distortion angles change periodically between adjacent domains. The resulting spontaneously formed homo‐materials superlattices imply that such ferroelectrics adopt domain‐specific Curie temperatures, in sharp contrast with the long‐held notion of *T*_C_ as a global materials parameter for a given ferroelectric.

## Graphical abstract

Temperature‐dependent electron diffraction on tin monochalcogenides demonstrates a gradual displacive transformation between ferroelectric and paraelectric phases with significant variability in lattice constants and Curie temperature (*T*_C_). Periodic variations exist even between domains in individual crystals, implying a domain‐specific Curie temperature in these layered ferroelectrics, in contrast to the long‐held notion of *T*_C_ as a global parameter for a given ferroelectric.

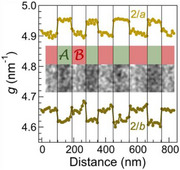

## 1 Introduction

Van der Waals ferroelectrics combine several unique attributes that are promising for device applications, including narrower bandgaps than traditional (oxide) ferroelectrics; reduced depolarization fields; in‐plane or out‐of‐plane polarization directions; and facile integration with a broad range of materials due to their layer structure and the possibility of using versatile transfer methods to build heterostructures [1]. Thin layered crystals also represent an attractive platform for implementing reconfigurable electronics [2, 3, 4, 5], where ordinary heterojunctions are complemented by domain walls [6] representing mobile interfaces that can be repositioned via external stimuli such as electric fields or strain. Fundamentally, van der Waals ferroelectrics promise to realize unconventional mechanisms and properties beyond those found in 3D crystals.

One of the most important attributes of both conventional 3D and 2D/van der Waals ferroics is the transformation between a polar low‐temperature phase and a high‐symmetry phase found above the Curie temperature, *T*_C_. This transformation generally falls in one of two major categories, namely displacive (diffusionless), relying on atomic motions smaller than the interatomic spacing; or order‐disorder, where the configuration of the atoms changes between an ordered state with aligned and a disordered state with randomly oriented dipoles (adding to a zero net polarization). Classic examples of displacive ferroelectrics [7] include BaTiO_3_ and LiNbO_3_ (although theory has suggested more complex behavior) [8, 9], whereas order‐disorder ferroelectrics include Rochelle salt [10] and potassium dihydrogen phosphate [11, 12].

Here, we investigate the nature of the phase transition for the ferroelectric van der Waals semiconductors SnS and SnSe. Synthetic few‐layer crystals of these tin monochalcogenides are layered ferroelectrics, showing a switchable electric polarization [13] and stripe domain patterns in piezoresponse force microscopy (PFM) [14, 15, 16, 17], polarized optical microscopy [6, 18], and electron microscopy/diffraction [18, 19]. While an anisotropic structure with built‐in (field switchable) electric dipoles implies that monolayer monochalcogenide crystals are invariably ferroelectric [20, 21], the origin of symmetry‐breaking and ferroelectricity in layered crystals has been unclear since the AB stacked orthorhombic equilibrium phase (space group *Pnma*) is centrosymmetric [22]. Prior work addressed (and mostly ruled out) several possible explanations, e.g., a net polarization due to an odd number of layers [14] or an alternative stacking order that breaks inversion symmetry [13, 16, 23, 24]. We recently demonstrated that ferroelectric SnS_1‐x_Se_x_ flakes across the entire composition range from SnS to SnSe adopt a nonequilibrium structure whose unit cell undergoes a small in‐plane distortion from the orthorhombic equilibrium lattice [19]. The distorted (monoclinic) polar structure transitions into a high‐temperature phase with a square in‐plane mesh at Curie temperatures ranging from 320°C (for SnSe) to ∼420°C (for SnS). In the present study, we use in situ nanobeam electron diffraction, performed on synthetic few‐layer SnS and SnSe flakes, to investigate this structural transition by tracking the evolution of the lattice between room temperature and *T*_C_ with nanometer spatial resolution. We find a displacive transformation in which the in‐plane lattice parameters (*a*, *b*) change continuously with increasing temperature until *a* = *b* at *T*_C_, which marks the transition to the symmetric high‐temperature phase. The transformation occurs over a wide temperature range, similar to the transition between the α‐ and β‐phase of bulk SnS(e), but the ferroelectric few‐layer crystals convert to the symmetric phase already at substantially lower temperatures. The lattice distortion, quantified by the angle γ between the in‐plane axes, also changes continuously with temperature until γ = 90° at *T*_C_. Strikingly, the thermally driven transition is not uniform, but lattice parameters and angles vary periodically between consecutive stripe domains in the polar phase. This implies that the Curie temperature is different for alternating domains. The concept of a domain‐specific Curie temperature breaks new ground in the field of ferroelectricity as it contrasts with the long‐held view of *T*_C_ as a global materials parameter for a given ferroelectric, with possibly far‐reaching consequences for functional and device properties. The findings highlight unique degrees of freedom in tin monochalcogenides that add to other unconventional properties of van der Waals ferroelectrics, such as moiré‐ or sliding ferroelectricity [25, 26, 27].

## 2 Results and Discussion

### 2.1 Stripe Domains in Ferroelectric Few‐Layer SnS and SnSe

**FIGURE 1:**
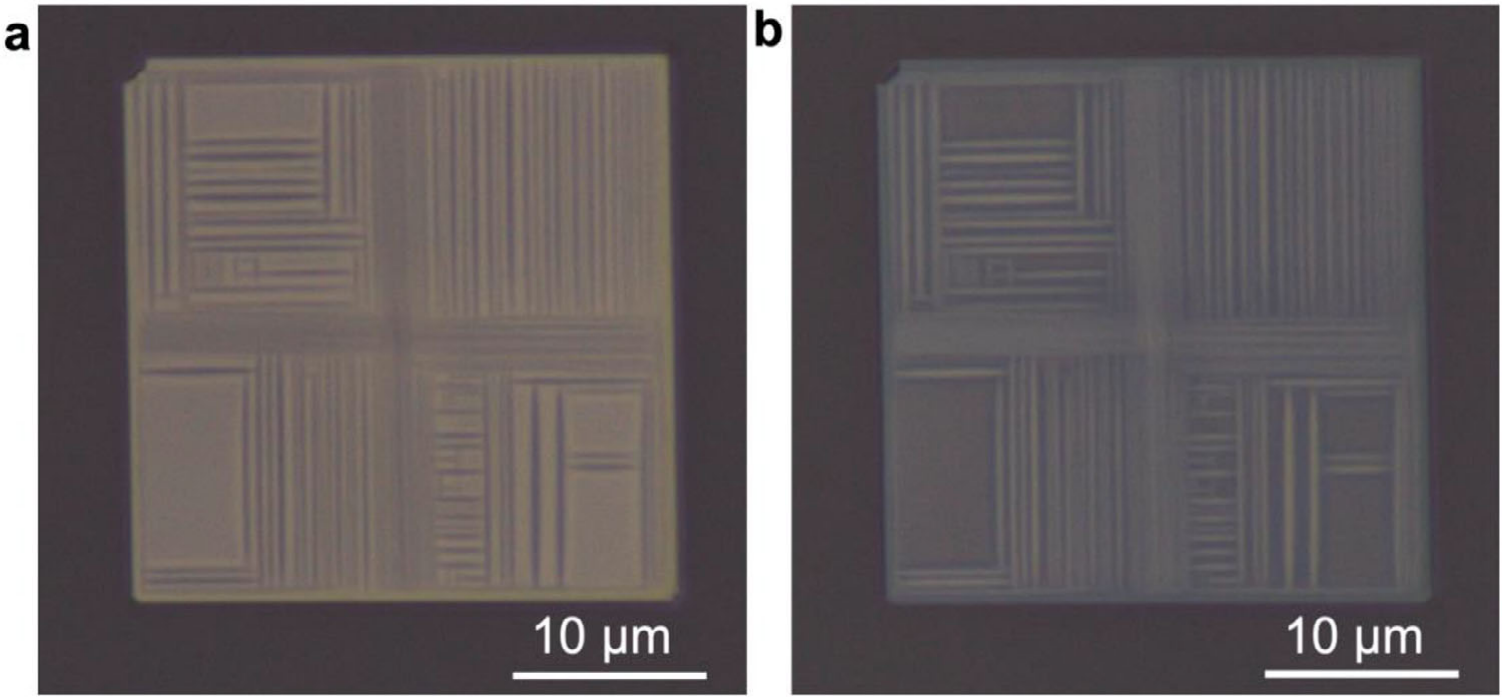
Polarized optical microscopy of stripe domains in ferroelectric few‐layer SnS flakes. (a). Polarized optical image of a synthetic SnS flake on the mica growth substrate, showing a pattern of in‐plane ferroelectric stripe domains. (b). The same ferroelectric crystal imaged with a complementary orientation of the analyzer axis, which gives rise to an inversion of the domain contrast.

Vapor transport growth from monochalcogenide (SnS, SnSe) powder precursors on mica van der Waals substrates yields ensembles of large crystalline few‐layer SnS(e) flakes (see Methods for details) [18]. For part of these ensembles (typically ∼10%–50% of the flakes), polarized optical microscopy shows stripe domain patterns. Figure 1 illustrates this optical contrast for an SnS crystal, where the contrast from the stripe pattern is inverted for complementary orientations of the reflected‐light analyzer. In prior work, we showed that the stripes—found for pure SnS and SnSe crystals, as well as SnS_1‐x_Se_x_ alloy flakes—are ferroelectric domains aligned along the in‐plane {110} axes and separated by mirror twin boundaries [18, 19, 28]. For the present study, such ferroelectric SnS and SnSe flakes were transferred to ultrathin (electron‐transparent) SiN_x_ membranes, on which transmission electron microscopy (TEM) and nanobeam electron diffraction (NBED) were performed at variable temperatures between room temperature and a maximum of ∼450°C, limited by the thermal decomposition of the layered crystals [29].

**FIGURE 2:**
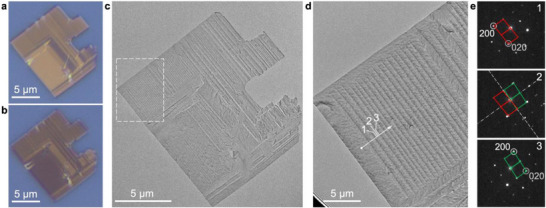
Transmission electron microscopy (TEM) of stripe domains in ferroelectric few‐layer SnS flakes at room temperature. (a,b). Polarized optical images, obtained with complementary analyzer orientations, of a synthetic SnS flake transferred to an electron‐transparent (20 nm) SiN_x_ membrane. (c). TEM image of the flake shown in panels (a), (b)., (d). Magnified view of the left corner of the flake (dashed rectangle in (c)), showing in‐plane ferroelectric stripe domains. (e). Nanobeam electron diffraction (NBED) data (part of a linescan of 100 diffraction patterns along the arrow in (d)), showing patterns obtained on either side of a domain wall (1,3) as well as at the domain wall (2), where both twinned patterns are visible.

Figure 2a,b. shows an example of a few‐layer SnS flake, transferred to a 20 nm SiN_x_ membrane and imaged using polarized optical microscopy, which shows a pattern of stripe domains aligned primarily along one of the {110} axes (i.e., parallel to one set of flake side facets). Figure 2c is a TEM image of the same crystal, which clearly shows domains even in areas where the width of the individual stripes is too narrow to be resolved by optical microscopy (see also Figure 2d). NBED allowed us to obtain diffraction patterns from such ferroelectric crystals at a spatial resolution substantially below the domain size. Figure 2e illustrates typical diffraction data obtained on a ferroelectric SnS flake at room temperature. Within the stripe domains, single‐crystal diffraction patterns are obtained (Figure 2e, Pos. 1, 3). At the intervening domain wall, diffraction shows a superposition of the patterns on either side (Figure 2e, Pos. 2), suggesting that the domain wall is a sharp twin boundary between mirrored monocrystalline domains [18]. The findings discussed in Figures 1 and 2 are characteristic of synthetic ferroelectric tin monochalcogenide flakes that crystallize across all compositions (i.e., SnSe_1‐x_S_x_ with 0≤ *x* ≤1) in a distorted nonequilibrium lattice structure, different from the orthorhombic equilibrium structure with space group *Pnma* [19]. To the best of our knowledge, such ferroelectric flakes have to date been obtained exclusively by synthesis on mica van der Waals substrates.

### 2.2 Analysis of the Phase Transition by Electron Diffraction at Variable Temperature

**FIGURE 3:**
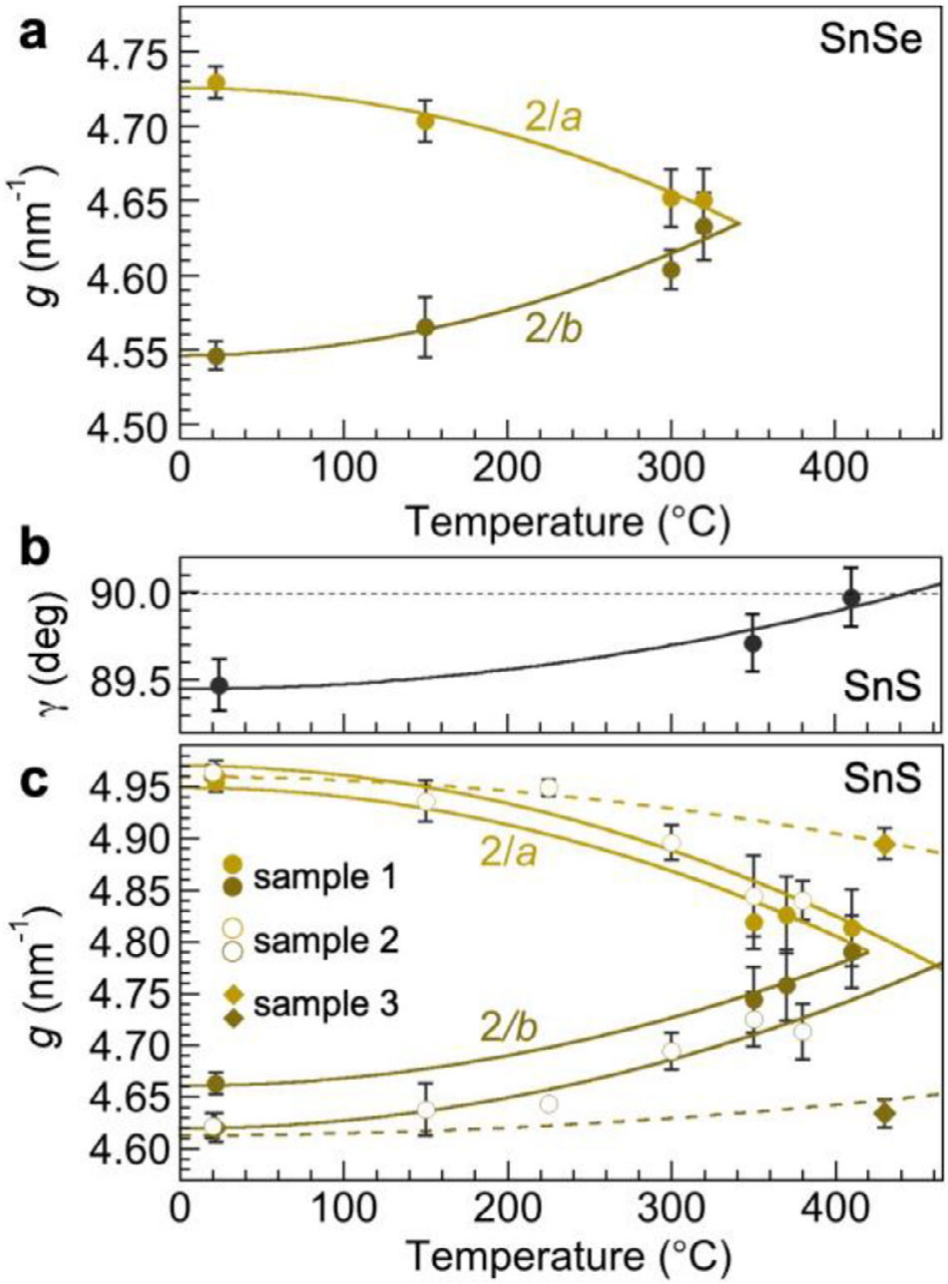
Temperature‐dependent nanobeam electron diffraction analysis of the displacive transition of ferroelectric few‐layer SnSe and SnS flakes during heating to *T*_C_. (a). Inverse lattice parameters (2/*a*,2/*b*) as a function of temperature measured on a synthetic ferroelectric SnSe flake. (b). Angle γ between the a^*^ and b^*^ reciprocal space axes measured from NBED linescans on a ferroelectric SnS flake at different temperatures. (c). Inverse lattice parameters as a function of temperature measured on three synthetic ferroelectric SnS crystals. Samples 1 and 2 are thin flakes with stripe domains; sample 3 is a thicker flake without any visible domains. Error bars in all plots represent ± one standard deviation of the averaged data. For underlying raw data, see (Data S10–S13 for panel (a); Data S1–S9 for panels (b,c)).

Nanobeam electron diffraction linescans analogous to Figure 2e were measured on ferroelectric SnSe and SnS flakes at variable temperatures to examine the evolution of the unit cell geometry between room temperature and *T*_C_. We focus here on changes in the lattice constants during heating. Effects observed during rapid and slow cooling, including the re‐formation of the stripe domain patterns following cooling from *T*_C_, were addressed in Ref. [19]. At each temperature, several NBED patterns were analyzed to obtain average inverse in‐plane lattice constants 2/*a* and 2/*b* and the angle γ between the reciprocal lattice vectors *a* ^*^ and *b* ^*^. The results are summarized in Figure 3. Figure 3a shows the evolution of 2/*a* and 2/*b* for SnSe (*T*_C_ ∼ 320°C) [18, 19]. The data illustrate the gradual nature of the transition, in which the lattice parameters, *a *= 0.423 nm and *b* = 0.440 nm at room temperature, continuously approach each other until they become equal at *T*_C_.

A similar analysis for two different SnS flakes is shown in Figure 3b,c. Figure 3b shows the temperature dependence of the angle γ between the reciprocal lattice vectors (*a* ^*^, *b* ^*^). Prior work demonstrated that the ferroelectric tin monochalcogenide phase is monoclinic, i.e., is distorted from the orthorhombic equilibrium phase for which γ = 90° [19]. This is confirmed by the diffraction analysis shown in Figure 3b, where γ = (89.47 ± 0.15)° at room temperature. Upon heating, the angle increases to γ = (89.97 ± 0.17)° at 410°C. Hence, attaining γ = 90° marks the transition to the paraelectric high‐temperature phase at *T*_C_ ≈ 420°C [19].

Figure 3c shows the evolution of the inverse in‐plane lattice parameters of two ferroelectric SnS flakes (samples 1, 2; for STEM images at different temperatures, see Figure S1), whose lateral size (25–50 µm) and thickness (10–20 nm) are consistent with recent observations for synthetic ferroelectric SnS [18]. The overall behavior is analogous to that found for SnSe, namely a gradual transition in which the in‐plane lattice constants (*a*, *b*) approach each other and become equal at *T*_C_. Generally, the behavior observed in Figure 3 for SnS and SnSe mirrors findings for the transformation between the low‐temperature α‐phase (space group *Pnma*) and the high‐temperature β‐phase (space group *Cmcm*) of bulk SnS(e), measured by neutron diffraction [30]. However, the transition occurs at substantially lower temperatures in our ferroelectric few‐layer flakes than in the corresponding bulk materials, where it is completed at ∼560°C and ∼630°C for SnSe and SnS, respectively (see the comparison shown in Figure S2).

Reference measurements performed on thicker (multilayer) SnS flakes without observable stripe domains in optical and electron microscopy, obtained in the same growth as the thin (ferroelectric) crystals (Figure 3c, sample 3), show γ = 90° as well as much smaller changes of the inverse in‐plane lattice parameters with increasing temperature, similar to the reported behavior of bulk SnS (with equilibrium lattice structure, space group *Pnma*) and consistent with prior experiments on thick SnSe_1‐x_S_x_ alloy flakes without visible stripe domains that found no significant changes in the (*a*, *b*) lattice parameters up to the onset temperature for thermal decomposition [19].

### 2.3 Domain Lattice Heterogeneity Probed by Spatially Resolved Nanobeam Electron Diffraction

**FIGURE 4:**
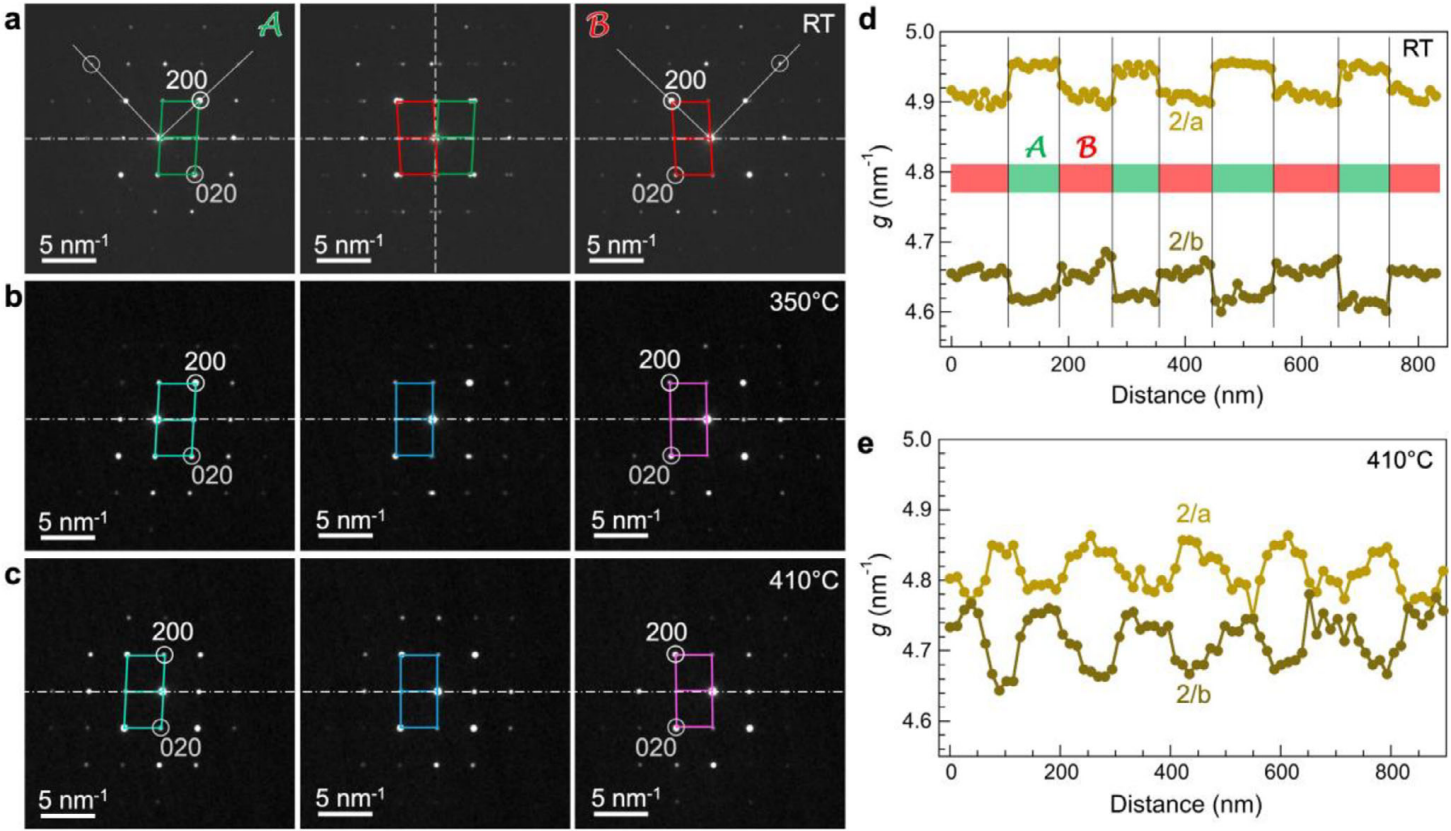
Temperature‐dependent nanobeam electron diffraction on a ferroelectric few‐layer SnS flake. (a). Nanobeam electron diffraction patterns obtained at room temperature (RT) in adjacent twin domains (left, green; right, red), and at the intervening domain wall (center). (b). Diffraction patterns obtained at 350°C on adjacent partially transformed stripe domains with reduced difference between the in plane (*a*, *b*) lattice parameters (cyan, purple), and *a* ≈ *b* at the location of the domain wall at room temperature (blue). (c). Diffraction patterns obtained at 410°C (i.e., close to T_C_) at the same positions as in (b), showing the late stage of the transformation. (d). Analysis of inverse lattice parameters (2/*a*, 2/*b*) from a linescan of 90 individual NBED patterns, obtained across 9 stripe domains (red, green) at room temperature. Note the different inverse lattice constants in neighboring domains. (e). Analysis of a linescan of 90 NBED patterns, obtained near T_C_ (410°C), showing nearly equal inverse lattice constants 2/*a* and 2/*b* in one set of almost transformed domains, while there is a persistent difference in the neighboring domains. For underlying raw data, see (Data S1 and S3).

We note that the average room temperature lattice parameters differ for the two analyzed SnS crystals, and the difference between 2/*a* and 2/*b* for the two samples persists as the temperature is increased, translating into different Curie temperatures, T_C_ ≈ 420°C (sample 1) and T_C_ ≈ 460°C (sample 2). This finding points to significant differences in the lattice constants of the as‐grown samples. According to Figure 3c, these differences are larger for *b* and quite small for *a*. This observed heterogeneity found in the ensemble averages of the measured lattice constants (as shown in Figure 3) prompted us to analyze the inverse lattice parameters with the full (sub‐domain scale) spatial resolution of our NBED linescans, again at different temperatures. Figure 4 summarizes the results for the example of sample 1.

Figure 4a displays room temperature NBED patterns on either side and at the location of the domain wall, showing the apparent twinning across the boundary. The diffraction patterns obtained at 350°C (Figure 4b) and 410°C (Figure 4c) correspond to partially transformed domains with an overall reduced difference between the in‐plane lattice constants *a* and *b*. Note the absence of a superposition pattern, found at room temperature, at the domain wall. Hence, at elevated temperatures, the abrupt switch in the diffraction patterns at a sharp domain wall is replaced by a gradual transition between the crystal structures in the domain interior so that *a* ≈ *b* at the former location of the domain wall (Figure 4b,c; center. See also Figure S5).

Figure 4d,e summarizes the analysis of the entire NBED linescans (across multiple domains) at room temperature and 410°C, respectively. The room temperature measurement shows that the heterogeneity in the inverse lattice constants, found in Figure 3 between different samples, is present at the domain level in individual ferroelectric crystals (Figure 4d). In fact, the NBED analysis shows differences in 2/*a* and 2/*b* between alternating A‐ and B‐type stripe domains. The different structure of consecutive domains is also reflected in the angle γ (see Figure S3), which assumes distinct values of 89.4° and 89.6° in A‐ and B‐domains, respectively. Note that there is no clear correlation between the domain‐to‐domain variations in lattice parameters or distortion angles and the width of the stripe domains (Figure 4d,e; Figures S3 and S4). At 410°C, the difference in lattice distortion – while still detectable – becomes small as the B‐domains have essentially reached the Curie transition (i.e., 2/*a* ≈ 2/*b*; γ ≈ 90°) while the A‐domains remain partially transformed and still show a sizable difference between 2/*a* and 2/*b*. Figure S5 illustrates the transition from the stripe domain pattern at room temperature to an essentially uniform lattice at 410°C. While there are clearly delineated, sharp domain walls at room temperature (Figure S5a,c), at temperatures near *T*_C_ the former domains are only identifiable via persistent differences in the inverse lattice parameters accompanied by small lattice rotations between consecutive stripes (Figure S5b,d). Given the gradual displacive transformation between the polar low‐temperature and the symmetric high‐temperature phase, the observed differences in the in‐plane lattice parameters, shown in Figure 4d,e, translate into sizable variations in Curie temperature between adjacent domains in the same ferroelectric SnS crystal (see Figure S6).

## 3 Conclusions

Our combined findings demonstrate that synthetic few‐layer tin monochalcogenide van der Waals ferroelectrics can show substantial variations in their room‐temperature lattice structure, consistent with a shallow energy landscape and a resulting lack of well‐defined ground‐state atomic positions [19]. The variability is especially pronounced along the *b*‐axis, likely owing to the puckered structure that allows the individual layers to easily accommodate substantial variations in *b*‐lattice parameter. The gradual displacive transformation to the symmetric high‐temperature phase (for which *a* = *b* and γ = 90°) implies that crystals with a larger difference Δ = (*b* – *a*) at room temperature will have a higher Curie temperature, *T*_C_.

Even more strikingly, coexisting in‐plane ferroelectric domains can adopt different lattice structures, i.e., can have domain‐specific Curie temperatures. Contrasting with the long‐held notion of *T*_C_ as a materials parameter that is constant for a given ferroelectric material, these characteristics represent a true paradigm shift in the field of ferroelectricity. While identifying the precise origin of the domain‐to‐domain lattice parameter variations will require further work, the available evidence points to a spontaneous organization during synthesis (which occurs at temperatures below *T*_C_, i.e., in the polar phase). Whereas a possible role of the substrate cannot be ruled out entirely, more likely explanations involve deformations in the process of stripe domain formation (with small energy penalties, see above), the need to match the lattices of adjacent domains at the {110}‐oriented boundaries, or distortions due to internal electric fields.

The different lattice parameters and Curie temperatures of consecutive stripe domains should also be detectable by means other than the temperature‐dependent structural characterization used here and manifest themselves in emerging functional properties of tin monochalcogenide van der Waals ferroelectrics. For example, one can anticipate that the domain‐specific *T*_C_ will give rise to unusual polarization‐temperature curves, reflecting the transition to the paraelectric phase in one set of domains while the ferroelectric phase persists in the complementary domains. Besides the observed thermal behavior, the self‐organization of the polar crystals into complementary stripe domains with alternating lattice structures carries potential for emerging functionality as in‐plane homo‐materials superlattices that may be reconfigurable by external stimuli such as electric fields or stain [31]. One can anticipate that the variable lattice constants will strongly affect the (electrically or elastically driven) domain wall motion, possibly stabilizing stripe domain patterns for applications such as domain wall electronics [2, 3, 4, 5]. More broadly, the unconventional van der Waals ferroelectrics discussed here can provide novel avenues for tuning functional characteristics such as electronic structure, charge conduction, or optoelectronic properties.

## 4 Materials and Methods

### 4.1 Materials Synthesis

Large few‐layer SnS and SnSe crystals were grown on mica substrates in a pumped 2‐inch quartz tube reactor with a single temperature‐controlled zone (MTI model OTF‐1200X). The growth used SnS (99.99%; Sigma–Aldrich) or SnSe (99.999%; ALB Materials) powders as precursors. The powders were finely ground using an agate mortar and pestle, placed in a quartz boat in the center of the evaporation zone, and heated to 580°C. Freshly exfoliated mica substrates (Ted Pella) supported by a refractory metal plate were placed with their leading edge 9 cm downstream from the source. During growth, an Ar (99.9999%; Matheson) carrier gas flow was maintained at 60 standard cubic centimeters per minute and a pressure of 20 mTorr. Growth was performed for 45 min, after which the reactor was cooled naturally to room temperature.

### 4.2 Electron Microscopy and Nanobeam Electron Diffraction

Transmission electron microscopy (TEM) was performed in a FEI Talos F200X microscope at 200 keV. SnS and SnSe crystals were transferred from mica growth substrates to chips carrying electron‐transparent SiN_x_ membrane windows (Simpore) by stamping on a four‐axis micromanipulator stage under optical microscope observation, using a polydimethylsiloxane (PDMS; Gelpak) elastomer assisted by deionized water to separate the crystals from the substrate. In situ electron microscopy and diffraction at variable temperature were performed on a Gatan 628 single‐tilt heating holder with Ta furnace and <0.1°C temperature stability. Domain patterns were imaged using TEM and scanning TEM (STEM). Nanobeam electron diffraction was performed in computer‐controlled linescans comprising linear arrays of equally spaced diffraction patterns exactly on [001] zone axis using an incident electron beam of ∼3 nm size.

### 4.3 Optical Microscopy

Polarized optical microscopy was performed in reflection geometry using an upright microscope (Olympus BX53) equipped with a fixed incident‐light polarizer and adjustable reflected‐light analyzer, 100× objective, and a high‐resolution (12.5 megapixel; Olympus DP75) scientific camera.

## Conflicts of Interest

The authors declare no conflicts of interest.

## Supporting information




**Supporting File 1**: advs73629‐sup‐0001‐SuppMat.docx.

**Supporting File 2**: advs73629‐sup‐0002‐Videos.zip.

## Data Availability

All data are available in the main text or the supporting information.
