# Supplementary material for: Displacive In‐Plane Ferroelectricity with Domain‐Specific Curie Temperature in Van der Waals Semiconductors
Source: Adv Sci (Weinh). 2026 Jan 4:e18341. Online ahead of print. doi: 10.1002/advs.202518341 (PMC13325564; doi:10.1002/advs.202518341)
Supplement: Supplementary file 1 — Supporting File 1: advs73629‐sup‐0001‐SuppMat.docx. [file ADVS-9999-e18341-s001.docx]

Supporting Information

Displacive In-Plane Ferroelectricity with Domain-Specific Curie Temperature in Van der Waals Semiconductors

Peter Sutter^1^*, Eli Sutter^2^

^1^Department of Electrical & Computer Engineering, University of Nebraska-Lincoln, Lincoln, Nebraska 68588, United States.

^2^Department of Mechanical & Materials Engineering, University of Nebraska-Lincoln, Lincoln, Nebraska 68588, United States.

*Corresponding author. E-mail: psutter@unl.edu

**Content of PDF file:**

Supporting Figures S1 to S6

**Other Supporting Materials for this manuscript include the following:**

Data S1 to S13

**Supporting Figures**


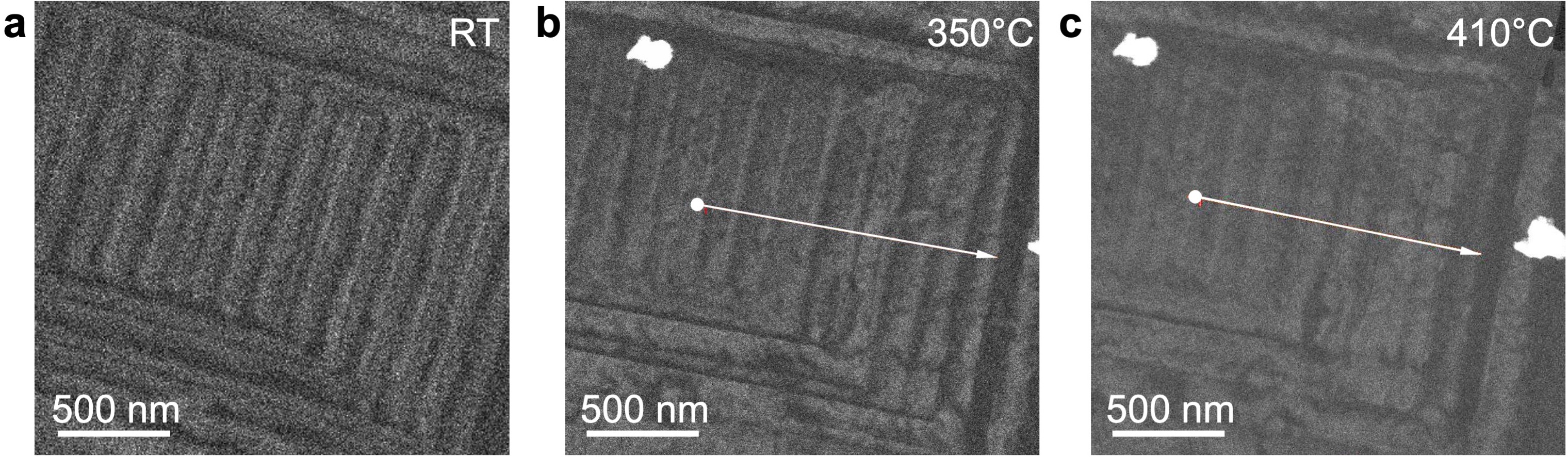


Figure S1. HAADF-STEM images of SnS stripe domains at different temperatures. a. Domain pattern at room temperature (RT). b. – c. Same sample area at 350°C (b.) and 410°C (c.), illustrating the evolution of the STEM contrast as *T*_C_ is approached.


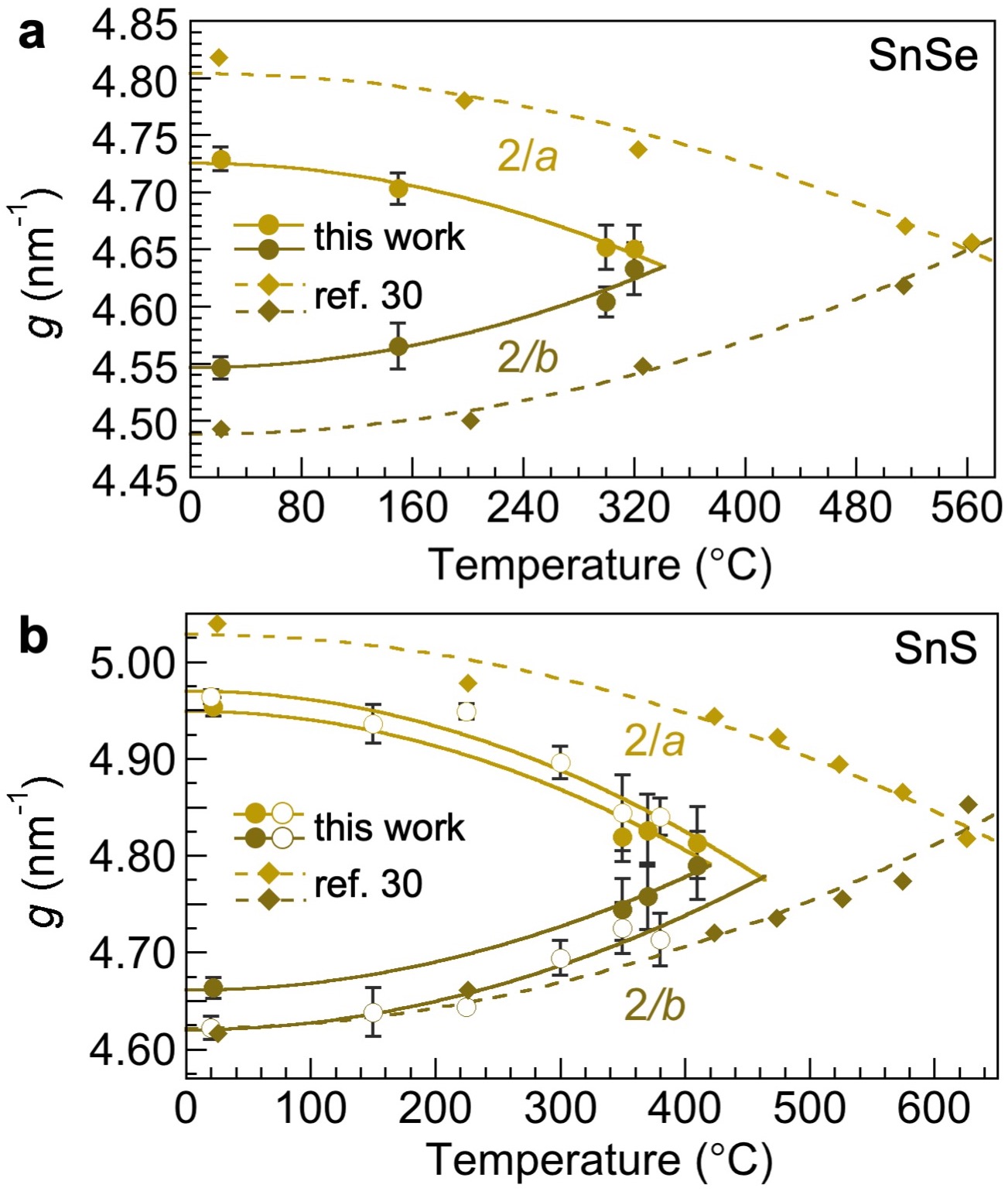


Figure S2. Temperature-dependent nanobeam electron diffraction analysis of the displacive transition of ferroelectric few-layer SnSe and SnS flakes during heating to T_C_. a. Inverse lattice parameters (2/*a*, 2/*b*) as a function of temperature measured on a synthetic ferroelectric SnSe flake, in comparison with the temperature dependent inverse lattice parameters of bulk SnSe (transition from α- to β-phase; Ref. 30 of the main text). b. Inverse lattice parameters as a function of temperature measured on two synthetic ferroelectric SnS flakes, in comparison with the temperature dependent inverse lattice parameters of bulk SnS (transition from α- to β-phase; Ref. 30 of the main text).


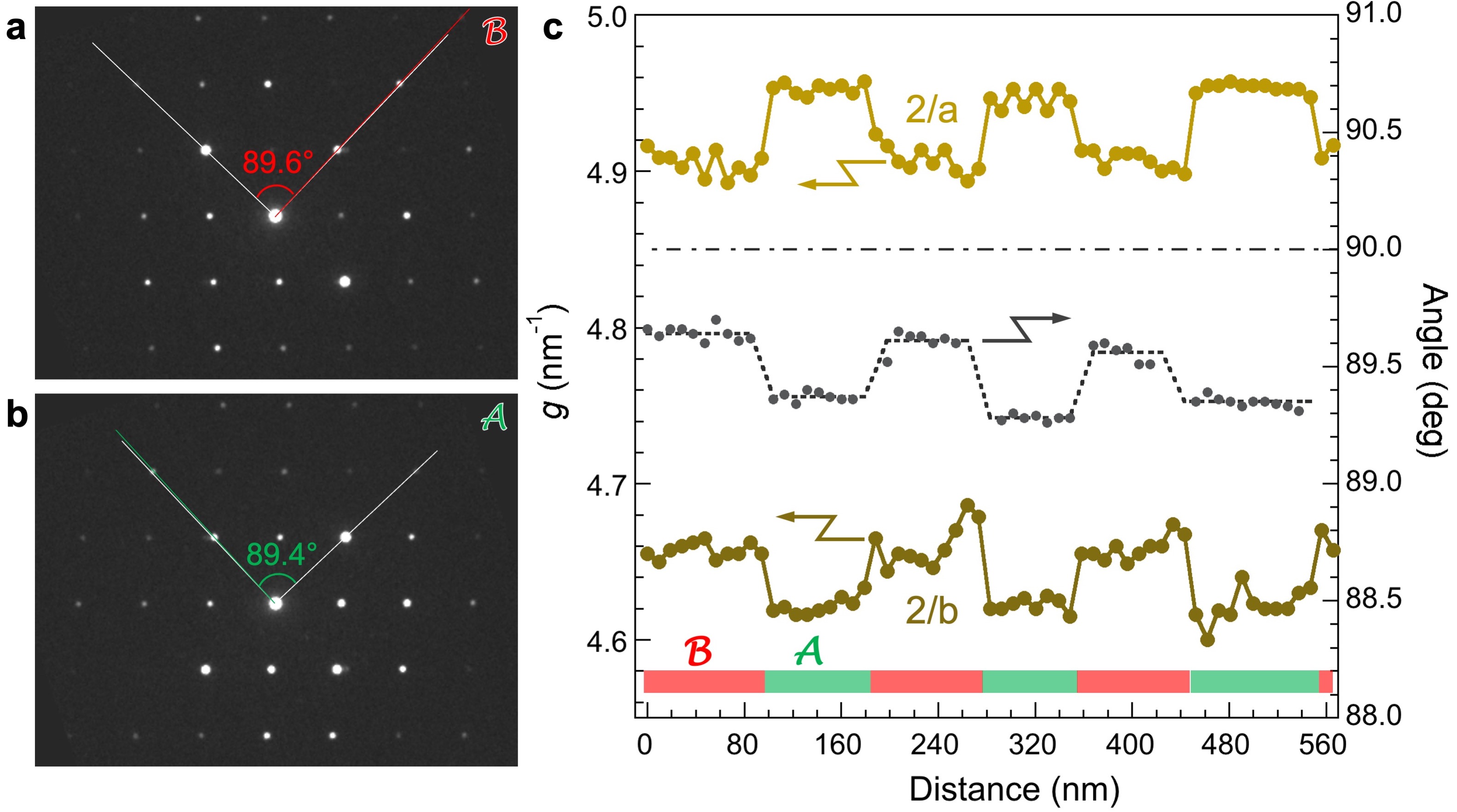


Figure S3. Angle between the *a*^*^ and *b*^*^ reciprocal axes for a ferroelectric SnS flake at room temperature. a. Nanobeam electron diffraction pattern of a ferroelectric B-domain, showing an angle γ = 89.6° between the *a*^*^ and *b*^*^ axes. b. Diffraction pattern of the complementary A-domain, showing an angle γ = 89.4°. c. Correlation between measured inverse lattice parameters (2/*a*, 2/*b*) and angle γ across 7 consecutive domains.


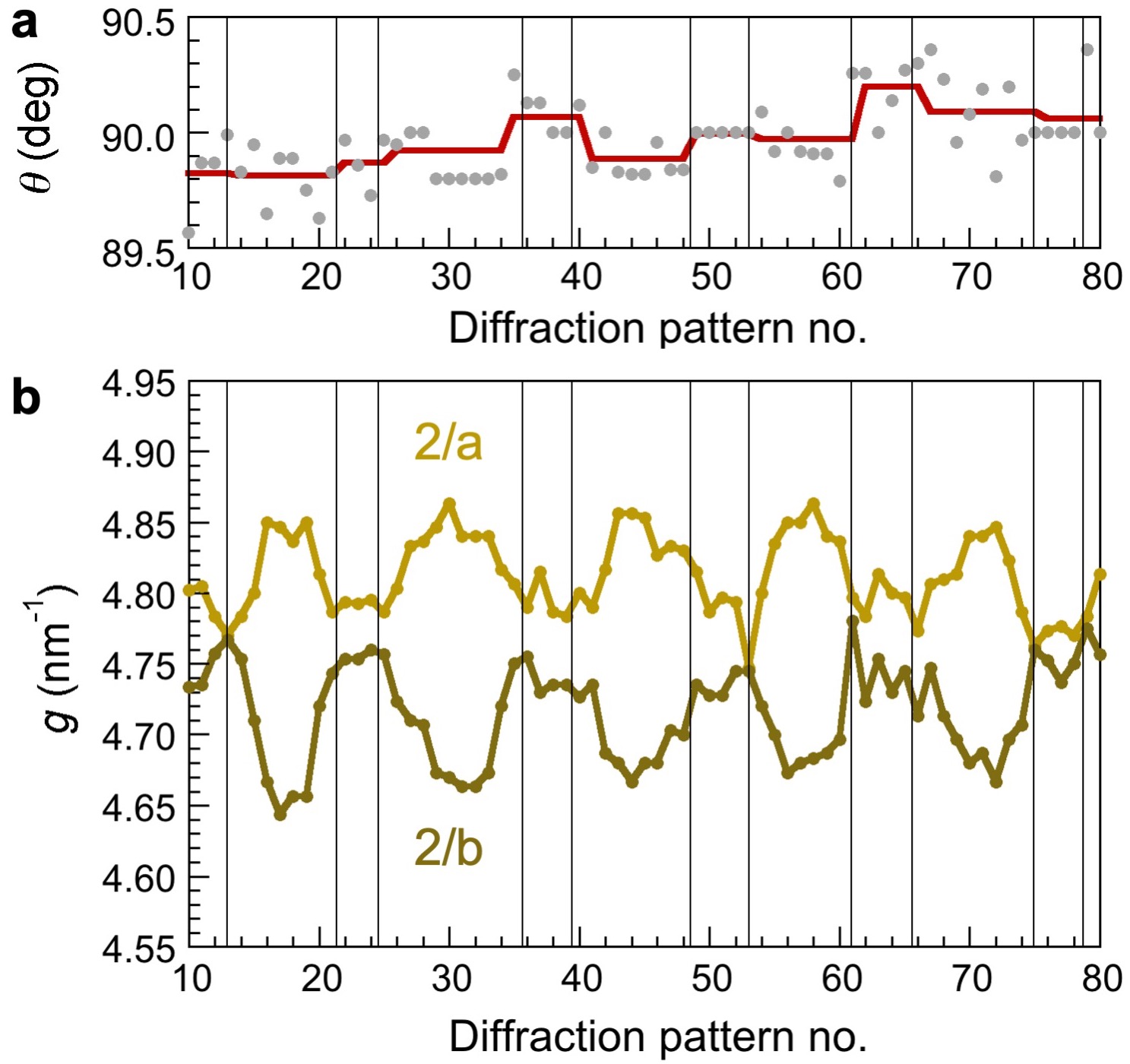


Figure S4. Correlation between the change in inverse lattice parameters and angle *γ* for an SnS flake near *T*_C_. a. Nanobeam electron diffraction analysis of the angle *γ* between the *a*^*^ and *b*^*^ reciprocal axes for a few-layer SnS flake at 410°C. b. Inverse lattice parameters determined from the same nanobeam electron diffraction linescan.


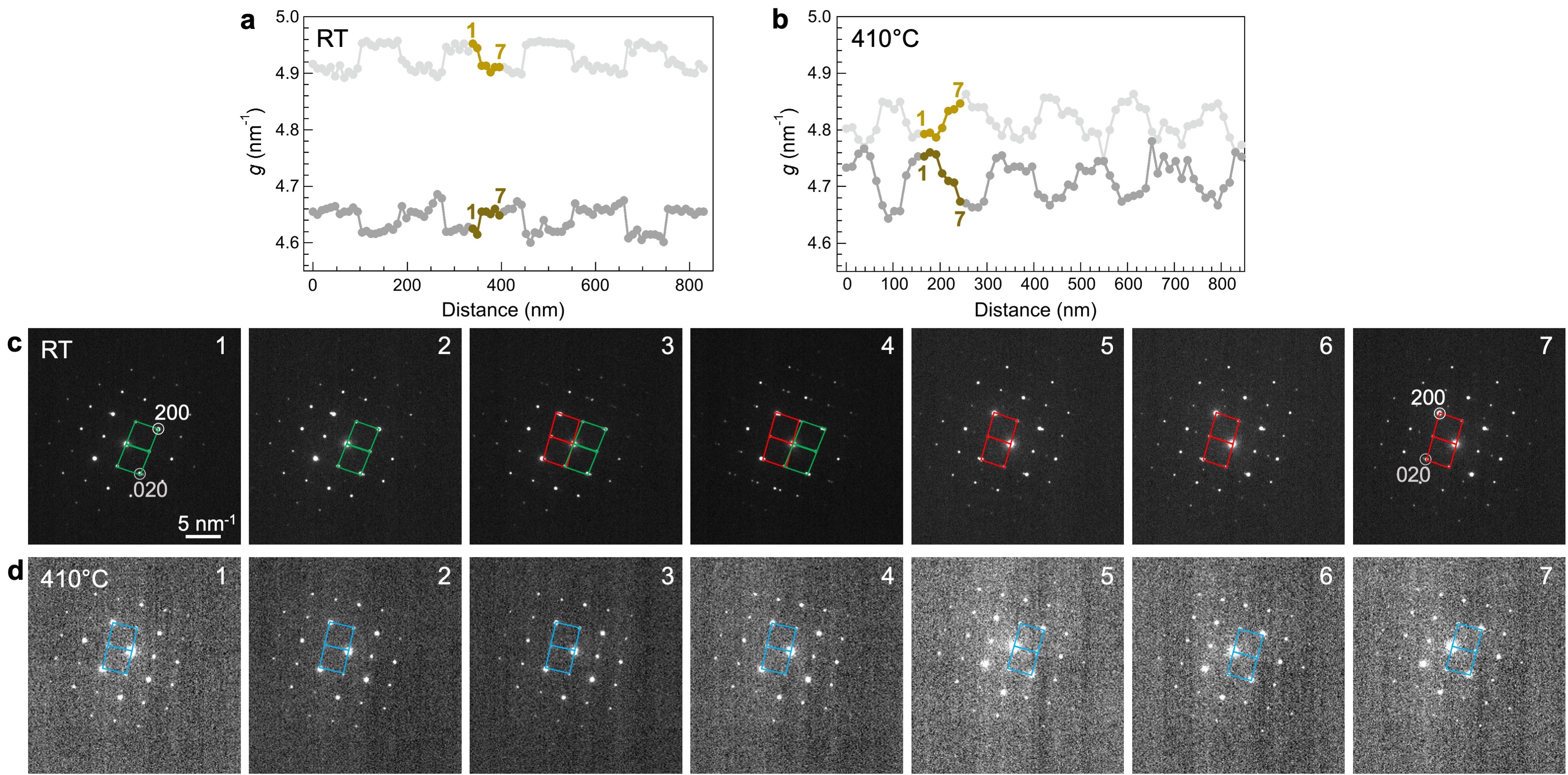


Figure S5. Loss of domain walls and persistence of lattice parameter differences at elevated temperatures. a. Analysis of inverse lattice parameters (2/*a*, 2/*b*) from a nanobeam electron diffraction linescan at room temperature (see Figure 4, main text). a. Analysis of inverse lattice parameters (2/*a*, 2/*b*) from a nanobeam electron diffraction linescan at room temperature (see Figure 4 d., main text). b. Analysis of inverse lattice parameters (2/*a*, 2/*b*) from a nanobeam electron diffraction linescan at 410°C (see Figure 4e., main text). c. Nanobeam electron diffraction patterns across one domain wall at room temperature (yellow/brown shaded portion of panel a.), showing twinning with abrupt transition between the two domains at the domain wall. d. Nanobeam electron diffraction patterns across the same distance at 410°C (yellow/brown shaded portion of panel b.), illustrating the loss of the domain wall but persistence of small differences between the lattice constants in adjacent domains.


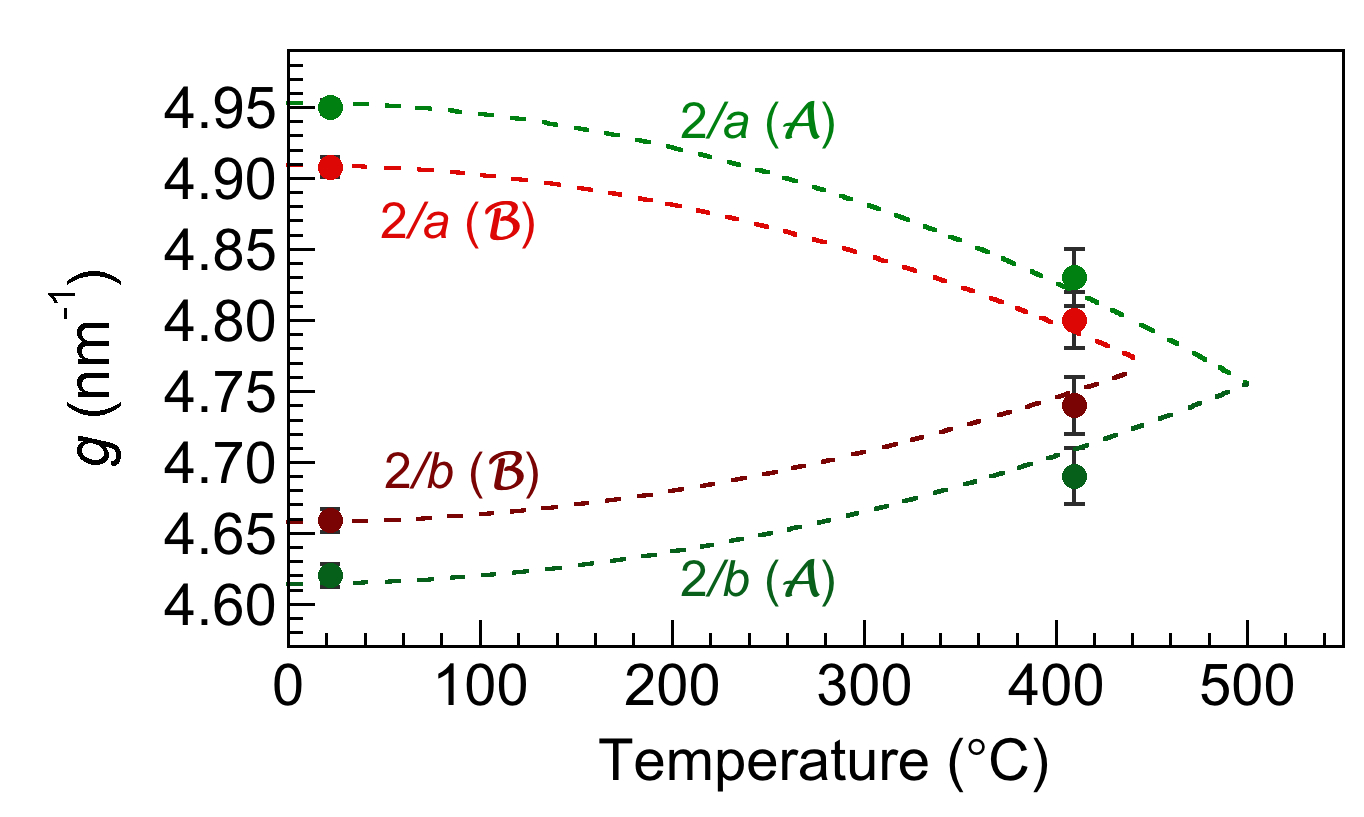


Figure S6. Domain-specific Curie temperatures. Inverse lattice parameters (2/*a*) and (2/*b*) for alternating A- and B-type stripe domains based on data shown in Figure 4 (main text). Dashed lines represent the same lineshape analysis shown for the average diffraction data (Figure 3, main text), whose intersection (*i.e.*, 2/*a* = 2/*b*) represents the transition to the square net characteristic of the high temperature paraelectric phase.

Data

Data S1.

Filename: “SnSSample1_RT.avi” File type: AVI movie.

Linescan of 100 nanobeam electron diffraction (NBED) patterns of a ferroelectric SnS crystal at room temperature (20240415 – Sample 1).

Data S2.

Filename: “SnSSample1_350C.avi” File type: AVI movie.

Set of 20 nanobeam electron diffraction (NBED) patterns of a ferroelectric SnS crystal at 350°C (20240415 – Sample 1).

Data S3.

Filename: “SnSSample1_410C.avi” File type: AVI movie.

Linescan of 100 nanobeam electron diffraction (NBED) patterns of a ferroelectric SnS crystal at 410°C (20240415 – Sample 1).

Data S4.

Filename: “SnSSample2_RT.avi” File type: AVI movie.

Set of 20 nanobeam electron diffraction (NBED) patterns of a ferroelectric SnS crystal at room temperature (20241016 – Sample 2).

Data S5.

Filename: “SnSSample2_150C.avi” File type: AVI movie.

Set of 20 nanobeam electron diffraction (NBED) patterns of a ferroelectric SnS crystal at 150°C (20241016 – Sample 2).

Data S6.

Filename: “SnSSample2_225C.avi” File type: AVI movie.

Set of 20 nanobeam electron diffraction (NBED) patterns of a ferroelectric SnS crystal at 225°C (20241016 – Sample 2).

Data S7.

Filename: “SnSSample2_300C.avi” File type: AVI movie.

Set of 20 nanobeam electron diffraction (NBED) patterns of a ferroelectric SnS crystal at 300°C (20241016 – Sample 2).

Data S8.

Filename: “SnSSample2_350C.avi” File type: AVI movie.

Set of 20 nanobeam electron diffraction (NBED) patterns of a ferroelectric SnS crystal at 350°C (20241016 – Sample 2).

**Data S9.**

Filename: “SnSSample2_380C.avi” File type: AVI movie.

Set of 20 nanobeam electron diffraction (NBED) patterns of a ferroelectric SnS crystal at 380°C (20241016 – Sample 2).

Data S10.

Filename: “SnSe_RT.avi” File type: AVI movie.

Set of 20 nanobeam electron diffraction (NBED) patterns of a ferroelectric SnSe crystal at room temperature (20240918 SnSe).

Data S11.

Filename: “SnSe_150C.avi” File type: AVI movie.

Set of 20 nanobeam electron diffraction (NBED) patterns of a ferroelectric SnSe crystal at 150°C (20240918 SnSe).

Data S12.

Filename: “SnSe_300C.avi” File type: AVI movie.

Set of 20 nanobeam electron diffraction (NBED) patterns of a ferroelectric SnSe crystal at 300°C (20240918 SnSe).

Data S13.

Filename: “SnSe_320C.avi” File type: AVI movie.

Set of 20 nanobeam electron diffraction (NBED) patterns of a ferroelectric SnSe crystal at 320°C (20240918 SnSe).
